# Temporal Parasitemia Trends Predict Risk and Timing of Experimental Cerebral Malaria in Mice Infected by *Plasmodium berghei* ANKA

**DOI:** 10.3390/pathogens14070676

**Published:** 2025-07-09

**Authors:** Peyton J. Murin, Cláudio Tadeu Daniel-Ribeiro, Leonardo José Moura Carvalho, Yuri Chaves Martins

**Affiliations:** 1 Department of Neurology, Saint Louis University School of Medicine, St. Louis, MO 63104, USA; peyton.murin@slucare.ssmhealth.com; 2 Laboratório de Pesquisa em Malária, Instituto Oswaldo Cruz and Centro de Pesquisa, Diagnóstico e Treinamento em Malária, Fundação Oswaldo Cruz, Rio de Janeiro 21040-360, RJ, Brazil; malaria@fiocruz.br (C.T.D.-R.); leojmc@ioc.fiocruz.br (L.J.M.C.); 3 Department of Anesthesiology, Saint Louis University School of Medicine, St. Louis, MO 63110, USA

**Keywords:** cerebral malaria, *Plasmodium berghei* ANKA, parasitemia dynamics, machine learning prediction, experimental mouse model

## Abstract

Background: Experimental models using *Plasmodium berghei* ANKA (PbA)-infected mice have been essential for uncovering cerebral malaria (CM) pathogenesis. However, variability in experimental CM (ECM) incidence, onset, and mortality introduce challenges when analyses rely solely on infection day, which may reflect different disease stages among animals. Methods: We applied machine learning to predict ECM risk and onset in a cohort of 153 C57BL/6, 164 CBA, and 53 Swiss Webster mice. First, we fitted a logistic regression model to estimate the risk of ECM at any day using parasitemia data from day 1 to day 4. Next, we developed and trained a Random Forest Regressor model to predict the exact day of symptom onset. Results: A total of 64.5% of the cohort developed ECM, with onset ranging between 5 and 11 days. Early increases in parasitemia were strong predictors for the development of ECM, with an increase in parasitemia equal to or greater than 0.05 between day 1 and day 3 predicting the development of ECM with 97% sensitivity. The Random Forest model predicted the day of ECM onset with high precision (mean absolute error: 0.43, R^2^: 0.64). Conclusion: Parasitemia dynamics can effectively identify mice at high risk of ECM, enabling more accurate modeling of early pathological processes and improving the consistency of experimental analyses.

## 1. Introduction

Malaria remains the most prevalent parasitic disease worldwide, with an estimated 263 million cases and 597,000 deaths reported in 2023 [1]. Among its complications, cerebral malaria (CM)—a severe manifestation of *Plasmodium falciparum* infection—continues to pose a significant global health challenge. CM is associated with high mortality rates (20–30%) and long-term neurocognitive impairments in up to 50% of survivors [2]. Despite decades of research, the pathogenesis of CM is not yet fully understood. There are currently no validated biomarkers or effective adjunctive therapies, highlighting the urgent need for better predictive and mechanistic insights [3,4].

Animal models, particularly the experimental cerebral malaria (ECM) model using *Plasmodium berghei* ANKA (PbA)-infected mice, have been instrumental in advancing our understanding of CM pathophysiology [5]. However, this model presents several limitations, including variability in ECM incidence (50–100%) and heterogeneity in the timing of onset, which ranges from day 5 to day 12 post infection [4,6,7,8,9]. In addition, factors such as the inoculum side, route of inoculation, number of parasite passages, and fact that the same mouse strain may differ depending on the origin may influence ECM outcome. In the present study, C57BL/6 and CBA mice were chosen due to their wide use and high susceptibility to ECM development (50–100% ECM incidence) [10]. We also included the host–parasite combination PbA-Swiss Webster described by our group due to its similarity in ECM presentation and susceptibility to both PbA-C57BL/6 and PbA-CBA models [11]. Furthermore, hallmark neurological symptoms—such as ataxia, convulsions, and coma—typically appear only hours before death, limiting the window for detecting early indicators of disease severity [12]. Consequently, relying on post-infection day as a surrogate for disease stage may introduce significant bias, as animals at the same time point may exhibit markedly different levels of disease progression [13].

Given that early prediction of ECM onset is critical for optimizing the timing of experimental interventions and therapeutic trials, efforts have been made to identify early markers of ECM development [6,11,13,14,15,16,17,18]. These studies have highlighted potential predictors such as neutrophil behavior, temperature, behavioral changes, and shifts in the T-cell receptor (TCR)-β repertoire. However, no existing study has developed a practical, predictive tool based solely on parasitemia data to anticipate ECM in individual animals before clinical deterioration.

In this study, we developed a supervised machine learning approach to predict ECM onset in PbA-infected mice using daily parasitemia measurements. We hypothesized that a model capable of integrating temporal changes in parasitemia could more accurately capture the complex dynamics underlying ECM development. Our approach offers a novel and accessible framework for reducing variability and bias in ECM studies, improving experimental design, and enhancing the identification of early pathophysiological changes. Moreover, a reliable method to forecast ECM development holds promise for increasing the translational value of this widely used animal model and deepening our understanding of CM pathogenesis.

## 2. Materials and Methods

### 2.1. Animals, Parasite, and Infection

This report was prepared according to the ARRIVE guidelines. Parasitemia data from 153 C57BL/6, 164 CBA, and 53 Swiss Webster mice was pooled from previous experiments performed at the Laboratory of Malaria Research, IOC-Fiocruz, Rio de Janeiro, Brazil. Infection and parasitemia measurement protocols were described before in detail [11,13]. Briefly, five- to eight-week-old female mice were obtained from ICTB-Fiocruz (Rio de Janeiro, Brazil), housed in groups of five maximum, and given free access to food and water. C57BL/6 and CBA mice weighed 20 ± 2 g at the time of infection. Swiss Webster mice weighed 26 ± 2 g at the time of infection. Mice were infected intraperitoneally with 1 × 10^6^ PbA-infected erythrocytes coming from a donor mouse of the same strain, age, and sex. Thin blood smears were made daily with a blood drop collected from the tip of the tail, stained according to the Panoptic method (Laborclin, Brazil) and examined under a light microscope (BH2, Olympus: Melville, New York, NY, USA) with an oil immersion lens (1000× final magnification). Parasitemia was determined by counting the number of parasitized RBCs in 2000 RBCs. Mice were observed three times a day and ECM diagnosis was made based on presentation of neurological clinical signs (ataxia, disorientation, paraplegia, roll-over, and coma) followed by death within 24 h after symptom development. Mice that did not develop ECM were euthanized 15 days post infection.

### 2.2. Data and Variables

The outcome variable in the study was the development of ECM from day 5 to day 11 post infection. The predictor variables consisted of parasitemia data from day 1 to day 4 post infection and mouse strain (C57BL/6, CBA or Swiss Webster).

### 2.3. Statistical Analysis

The statistical plan was devised prior to accessing the data. All analysis was performed using GraphPad Prism Version 10.5.0 (GraphPad Software. Boston, MA, USA) or Python Version 2.3.1. (Anaconda Software Distribution. Austin, TX, USA). A log-rank test was used to compare survival curves. The following add-ons were used for analysis: pandas [19], numpy [20], itertools [21], sklearn.model_selection, sklearn.ensemble, sklearn.linear, sklearn.metrics, sklean.preprocessing, sklearn.impute [22], seaborn [23], and matplotlib.pyplot [24].

### 2.4. Imputation of Missing Predictor Values

All predictors in the analytic datasets were continuous numeric variables. We dealt with sporadic missing variables using univariate mean imputation, implemented with scikit-learn’s SimpleImputer (strategy = “mean”). Imputation performed separately for each modeling task (Early-onset-CM regression (X_regression), Imminent-risk” classification (X_classification, Exact-day regression (X_imminent_day)). For each individual task, the imputer was fitted on the training partition (i.e., the column-wise arithmetic mean was estimated from the non-missing values of the training set). The fitted imputer was then applied unchanged to the test set, limiting any information leakage.

### 2.5. Outcome Variables

The target variable had no missing value and was, therefore, not imputed.

### 2.6. Rationale

Mean substitution was chosen because (i) the proportion of missingness was low, (ii) all predictors were approximately symmetric and on continuous scales, and (iii) there was no evidence that the likelihood of missing variables associated with the outcome variables.

Then, a logistic regression was performed as follows:

Let Y_ij_ denote the development of ECM (1 = ECM; 0 = no ECM) for the ith participant at the ith time point, where Y_ij_ = 1 indicates the presence of the event and Y_ij_ = 0 indicates its absence. For each participant i, the response Y_ij_ is observed over a series of day pairs (t_1_, t_2_).

logit(P(Y_ij_ = 1)) = β_0_ + β_1_ΔP_ij_ + b_i_

where

(P(Y_ij_ = 1)) is the probability that the outcome occurs for participant i at day pair j.

ΔP_ij_ = P_ij_(t_2_)−P_ij_(t_1_) represents the parasitemia change between two time points.

β_0_ is the intercept term.

β_1_ is the coefficient capturing the effect of parasitemia change.

b_i_ ∼ N(0,σ2) is a participant-specific random effect to account for within-subject correlation.

Optimal parasitemia threshold τ for parasitemia change is identified by maximizing the average precision (AP) score:

τ = argτ max AP (Y, Ŷ (ΔP ≥ τ))

where Ŷ is the predicted outcome based on whether the parasitemia change exceeds the threshold:

Ŷ_ij_ = 1, if ΔP_ij_ ≥ τ

Ŷ_ij_ = 0, if ΔP_ij_ < τ

Resultant probability was calculated for each day pair (t_1_, t_2_) and threshold value τ, as follows:

P(Y_ij_ = 1) = σ(β0 + β1 (ΔP_ij_−τ))

The performance of the logistic regression model was assessed using sensitivity, specificity, AP, recall, area under the precision recall curve (PR-AUC), and f1-score for each day pair.

We also hypothesized that a limitation of the model could be differences between strains. To assess this, the data was split by strain, and analysis was repeated as described above. Model performance was again assessed for each day pair for each strain.

### 2.7. Random Forest Regressor Model

Although our initial analysis focused solely on predicting the occurrence of ECM as a binary outcome (1 or 0), we later hypothesized that the dynamics of parasitemia could be leveraged to predict the specific day on which ECM onset occurs. Using pandas, data was uploaded with the columns ‘ID’, ‘Strain’, day of ECM onset, and daily parasitemia data for day 1 to day 10. The data was reshaped into a long format, the target variable was defined, feature engineering was used to create a “Parasitemia_Change” column and “Parasitemia_MA3” rolling mean of the 3 days prior to ECM onset. The data was saved for modeling. Features were defined as “Parasitemia”, “Parasitemia_Change”, and ‘Parasitemia_MA3’, with the target variable defined as “CM_Risk.” Data was split into training and testing sets with a split of 80% training, 20% testing, and a random state of 42. Any missing data was imputed using the sklearn SimpleImputer (version 1.7.0). The model was initialized using rf_model with 100 estimators, a random state of 42, and a balanced class weight. The model was fitted using rf_model.fit and used to make predictions using rf.model.predict. Performance was assessed using classification_report with precision, recall, f1-score, and support. To optimize model performance, sklearn GridSearchCV (version 1.7.0) was used to identify optimal model parameters based upon best mean square error (MSE). Performance was then assessed using the MSE, R2, mean absolute error (MAE), and cross-validated MSE, and the learning curve was plotted.

## 3. Results

### 3.1. ECM Incidence and Parasitemia Dynamics

**Figure 1:**
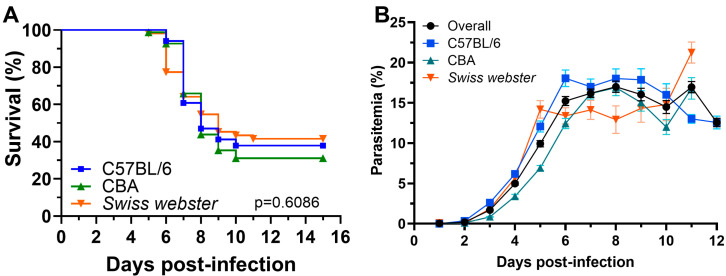
(**A**) Kaplan–Meier survival plot showing the percentage of mice who had developed experimental cerebral malaria (ECM) by each day. All mice who would develop ECM had done so by day 11. A log-rank test was used to compare survival curves. (**B**) Line plot showing the temporal dynamics of parasitemia. The dots represent the average parasitemia, in percentages, at each time point and the error bars represent the standard error of the mean.

The overall incidence of experimental cerebral malaria (ECM) was 64.5% (64% in C57BL/6, 69% in CBA, and 51% in Swiss Webster mice). Survival curves did not significantly differ between strains. Mice developed ECM between days 5–11 post infection, with strain-specific ranges: 6–10 days in C57BL/6, 5–10 in CBA, and 5–11 in Swiss Webster (Figure 1A).

Parasitemia levels increased most rapidly between days 1 and 3, rising from a mean of 0.04% to 1.69%. This was followed by a more gradual increase from days 4 to 6, reaching 15.22% from an initial mean 4.98%. Between days 7 and 9, parasitemia plateaued, remaining relatively stable (mean parasitemia from 16.19% to 16.03%), before gradually declining from 14.49% to 12.57% between days 12 and 14 in the mice that survived (Figure 1B).

### 3.2. Logistic Regression Model: Cohort-Level Analysis

We first trained a logistic regression model on the full dataset (all strains combined) to predict ECM risk from early parasitemia changes. This resulted in optimal changes in parasitemia of −0.05% between day 1 and day 3 (AP: 0.67), 0.16% between day 1 and day 4 (AP: 0.67), 0.12% between day 2 and day 3 (AP: 0.66), 0.14% between day 2 and day 4 (AP: 0.67), and 2.34% between day 3 and day 4 (AP: 0.66). The resultant probability of CM was the highest for a change of 0.05% or greater between day 1 and day 3 (93%). To assess model performance, precision, recall/sensitivity, specificity, and f1-score were calculated for each day pair. Sensitivity exceeded 90% across several day pairs (days 1–3, 1–4, 2–3, and 2–4), while specificity was modest, peaking at 0.43 for days 3–4. Precision and f1-score values were highest for day pair day 1 to day 3 (precision: 0.67, f1-score: 0.79), day 1 to day 4 (precision: 0.67, f1-score: 0.79), and day 2 to day 4 (precision: 0.67, f1-score: 0.79) (Table 1). The model was converted into a usable tool within RedCap (publicly available at https://redcapsurvey.slu.edu/surveys/?s=7NH7FR3FRN8D3ERW, accessed on 3 July 2025), which can identify the likelihood of a mouse developing ECM, based upon parasitemia data from day 1 to day 4.

### 3.3. Strain-Specific Logistic Regression

To assess if performance could be improved considering lineage, we took a two-fold approach. First, the model was trained with strain included; however, overall performance was minimally impacted by the inclusion of this variable. This resulted in optimal changes in parasitemia of 0.05% between day 1 and day 3 (AP: 0.67, f1-score: 0.77), 0.16% between day 1 and day 4 (AP: 0.67, f1-score: 0.79), 0.12% between day 2 and day 3 (AP: 0.66, f1-score: 0.76), 0.14% between day 2 and day 4 (AP: 0.67, f1-score: 0.79), and 2.34% between day 3 and day 4 (AP: 0.66, f1-score: 0.74). Alternatively, we assessed if performance could be improved when the mice were separated by strain. Overall performance was again minimally impacted. The highest AP for any day pair was 0.68 in the C57BL/6 mice cohort, 0.72 in the CBA cohort, and 0.60 within the Swiss Webster cohort. The highest f1-score was 0.79 in the C57BL/6 mice cohort, 0.83 in the CBA cohort, and 0.67 in the Swiss Webster cohort. The cutoffs values for all the respective day pair–strain combinations and their respective performance parameters can be seen in Table 1.

### 3.4. Predicting Day of ECM Onset with Random Forest Regression

**Figure 2:**
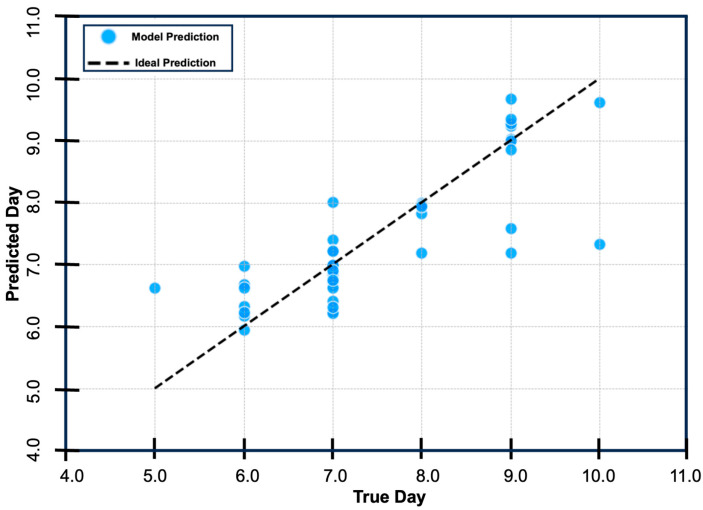
Learning curve for the Random Forest Regressor model. The *y*-axis represents the predicted day of experimental cerebral malaria (ECM) onset. The *x*-axis represents the true day of ECM onset. The dotted line represents perfect model performance. The blue dots represent the distribution of the actual datapoints within the test set. The model displayed excellence performance with a cross-validated MSE = 0.2924 ± 0.1461.

As a secondary step, we sought to train and fit a Random Forest Regressor model to predict the day of ECM onset using available parasitemia data from the preceding days. The model takes all available parasitemia data (from day 1 through day 11), imputes any future values or missing values, and provides a precise estimate of the day of ECM onset. Importantly, the model predicted the day of onset of ECM within 0.54 ± 0.38 days across the test dataset, with a cross-validated MSE = 0.2924 ± 0.1461, R2 = 0.6378, and MAE = 0.4301 (Figure 2). To facilitate the broader application of our model in ECM research, we developed an extended version that enables users to input parasitemia measurements and obtain a predicted day of ECM onset. The model accepts parasitemia data from days 1 through 11 of infection. To accommodate incomplete datasets, we incorporated an imputation algorithm that permits accurate prediction based on as few as two data points (e.g., parasitemia levels from days 1 and 3). The random forest model, the imputer, and the predictor extension are publicly available at https://github.com/pmurin29/cm-risk-prediction, accessed on 3 July 2025.

## 4. Discussion

An ideal model for the identification of ECM would (1) identify high-risk mice before the onset of neurological signs (prior to day 5), (2) predict the approximate day of onset facilitating timing of interventions and accurate comparison of disease stages, and (3) be easily applied without the need for time-consuming tests or complex assays. In the present study, we applied supervised machine learning, in the form of a multivariable logistic regression, to predict the development of ECM in mice based on parasitemia dynamics from day 1 to day 4. This resulted in parasitemia change threshold values predicting the onset of ECM with near perfect sensitivity at multiple day pairs, achieving aim 1 and aim 3. However, logistic regression models are limited to binary outcome predictions, making it ineffective to predict day of onset. To overcome this limitation, we applied supervised machine learning in the form of a Random Forest Regressor to determine if we could predict the exact day of ECM, achieving robust performance, with the model predicting the day of ECM within 0.54 ± 0.38 days across the dataset (MSE: 0.2924 ± 0.1461), thereby achieving aims 1–3. Therefore, we demonstrated that parasitemia levels can be an efficacious and robust predictor for the development of ECM in PbA-infected mice.

There have been several previous attempts to address the challenge of predicting ECM onset in mice. A symptom-based approach using the SHIRPA protocol was able to predict the development of ECM with a high positive predictive value (>89%) on days 5 or 6 post infection [13,25]. However, such a model requires a time-consuming and skilled assessment and is limited in its ability to detect ECM earlier than 24 h before the onset of clinical signs [26]. Several biomarker-based approaches have been utilized. In a study of neutrophil dynamics in PbA-infected mice, a higher percentage of neutrophils was noted in the spleen and peripheral blood in ECM-susceptible C57BL/6 mice compared to ECM-resistant BALB/c mice at day 4 post infection [15]. However, while these differences suggest that neutrophils may offer potential as a biomarker for ECM, it is unclear if these preliminary findings could be utilized to predict ECM development and/or the time of onset. In a study of T-cell receptor signature alterations in mice infected with PbA, alterations in the T-cell receptor repertoires were noted in the spleen and peripheral blood on day 5 and day 6 for mice with ECM compared to mice without ECM [17]. This is supported by the well-established role of T-cells in ECM and malaria pathogenesis [27,28,29]. However, such an approach is again limited in its ability to predict ECM early in the course of the disease, prior to ECM onset [26].

Our study was not without limitations. Not all parasitemia data was available for all days, requiring the use of imputation. While the logistic regression model was highly sensitive for the identification of ECM, specificity was limited. Not all mice were of the same strain. While strain was considered in the development of the logistic regression model, the inclusion of strain as a covariate did not appear to improve performance. Given the small sample sizes within individual strains and the associated risk of overfitting, we did not attempt to train the Random Forest model by strain. Therefore, although unlikely, the performance of the Random Forest model may be lower in the Swiss Webster mice than the one reported by us. Future work will focus on further training and refining the Random Forest model to enhance predictive accuracy. Additionally, previous studies have indicated that ECM may exist as a spectrum [30,31], and the misclassification of mice that developed some degree of neurobehavioral symptoms may have occurred in our study. Although this is a limitation of our work, it does not impact its main goal of developing an easy tool to predict mice at the highest risk for ECM development. Further studies trying to determine the association of parasitemia levels with different features of the ECM phenotypic spectrum such as blood–brain barrier disruption levels are needed to better understand the disease pathophysiology.

## Figures and Tables

**Table 1:** Logistic regression model performance.

Overall Cohort	Best Threshold	Precision	Recall	Sensitivity	Specificity	F1-Score	PR-AUC
Day 1 to Day 2	0.01	0.66	0.83	0.83	0.21	0.74	0.66
Day 1 to Day 3	0.05	0.67	0.97	0.97	0.14	0.79	0.67
Day 1 to Day 4	0.16	0.67	0.95	0.95	0.15	0.79	0.67
Day 2 to Day 3	0.12	0.66	0.90	0.90	0.17	0.76	0.66
Day 2 to Day 4	0.14	0.67	0.95	0.95	0.16	0.79	0.67
Day 3 to Day 4	2.34	0.67	0.64	0.64	0.43	0.65	0.66
C57BL/6							
Day 1 to Day 2	0.08	0.71	0.50	0.50	0.64	0.59	0.68
Day 1 to Day 3	0.04	0.67	0.95	0.95	0.18	0.79	0.67
Day 1 to Day 4	0.16	0.67	0.94	0.94	0.16	0.78	0.67
Day 2 to Day 3	0.09	0.67	0.88	0.88	0.22	0.76	0.66
Day 2 to Day 4	0.14	0.67	0.94	0.94	0.16	0.78	0.67
Day 3 to Day 4	2.73	0.69	0.49	0.49	0.60	0.57	0.66
CBA							
Day 1 to Day 2	0.01	0.71	0.86	0.86	0.18	0.77	0.67
Day 1 to Day 3	0.07	0.72	0.98	0.98	0.14	0.83	0.74
Day 1 to Day 4	5.08	0.75	0.44	0.44	0.66	0.55	0.73
Day 2 to Day 3	0.24	0.72	0.85	0.85	0.26	0.78	0.74
Day 2 to Day 4	5.13	0.75	0.43	0.43	0.68	0.55	0.74
Day 3 to Day 4	9.42	1.00	0.07	0.07	1.00	0.13	0.72
Swiss Webster							
Day 1 to Day 2	0.27	0.58	0.56	0.56	0.58	0.57	0.48
Day 1 to Day 3	4.68	1.00	0.15	0.15	1.00	0.26	0.63
Day 1 to Day 4	3.86	0.63	0.70	0.70	0.58	0.67	0.64
Day 2 to Day 3	3.89	1.00	0.15	0.15	1.00	0.26	0.64
Day 2 to Day 4	4.06	0.64	0.67	0.67	0.62	0.65	0.66
Day 3 to Day 4	3.14	0.63	0.63	0.63	0.62	0.63	0.64

PR-AUC = area under the precision recall curve.

## Data Availability

The original data presented in the study are openly available on Zenodo at doi: 10.5281/zenodo.15557233.

## Abbreviations

The following abbreviations are used in this manuscript:

AP	Average Precision
ARRIVE	Animal Research: Reporting of In Vivo Experiments (guidelines)
CNPq	Conselho Nacional de Desenvolvimento Científico e Tecnológico (Brazil)
CM	Cerebral Malaria
ECM	Experimental Cerebral Malaria
Faperj	*Fundação Carlos Chagas Filho de Amparo à Pesquisa do Estado do Rio de Janeiro*
Fiocruz	*Fundação Oswaldo Cruz*
ICTB	*Instituto de Ciência e Tecnologia em Biomodelos*
INCT-NIM	*Instituto Nacional de Ciência e Tecnologia em Neuroimunomodulação*
IOC	*Instituto Oswaldo Cruz*
LPM	*Laboratório de Pesquisa em Malária*
MAE	Mean Absolute Error
MSE	Mean Squared Error
PbA	*Plasmodium berghei* ANKA
PR-AUC	Precision-Recall Area Under the Curve
R^2^	Coefficient of Determination (R-squared)
RBC	Red Blood Cell
RedCap	Research Electronic Data Capture
SHIRPA	SmithKline Beecham, Harwell, Imperial College, Royal London Hospital, Phenotype Assessment (protocol)
TCR	T-cell Receptor
